# Criterion Validity and Reliability of SF-12 Health Survey Version 2 (SF-12v2) in a Student Population during COVID-19 Pandemic: A Cross-Sectional Study

**DOI:** 10.1155/2021/6624378

**Published:** 2021-08-04

**Authors:** Ilaria Ruotolo, Anna Berardi, Giovanni Sellitto, Francescaroberta Panuccio, Antonella Polimeni, Donatella Valente, Giovanni Galeoto

**Affiliations:** ^1^Sapienza University of Rome, Italy; ^2^Department of Human Neurosciences, Sapienza University of Rome, Italy; ^3^Department of Odontostomatological and Maxillo Faccial Sciences, Sapienza University, Rome, Italy; ^4^IRCSS Neuromed, Via Atinense, 18, 86077 Pozzilli, Italy

## Abstract

**Objective:**

The novel coronavirus SARS-CoV-2, responsible for the COVID-19 pandemic, led to strict domestic quarantine, social isolation policies, and consequently significant psycho-emotional and lifestyle changes. The individual and societal fear and anxiety cause significant stress affecting health-related quality of life (HRQOL). There is evidence of the psychological and mental health effects of the current pandemic on students, who are known to be a vulnerable population. A decrease in physical activity was reported among students, and it is known to contribute to stress levels, which is strongly associated with HRQOL. This study is aimed at evaluating the validity and reliability of SF-12 Health Survey version 2 (SF-12v2) in the assessment of self-perceived quality of life of Italian students following lifestyle changes due to the outbreak of COVID-19.

**Methods:**

A cross-sectional study was conducted with university students attending the faculty of Medicine and Surgery or Health Professions. The Physical Component Summary (PCS12) and the Mental Component Summary (MCS12) of SF-12v2 were compared to the Perceived Stress Scale (PSS). Internal consistency was examined using Cronbach's Alpha Coefficient. Concurrent validity was evaluated comparing SF-12v2 values to PSS scores, and the Pearson Correlation Coefficient (PCC) was calculated. Cross-cultural validity was investigated through several analyses for correlations between SF-12v2 scores and the gender of participants, University of Italy, body mass index (BMI), and time spent sitting and exercising.

**Results:**

The SF-12v2 questionnaire was administered to 583 medical and health professionals' students in July 2020. Cronbach's Alpha showed acceptable reliability for PCS12 and MCS12. In line with expectations, PCS12 scores differed by BMI groups, while the MCS12 was associated with PSS score and showed differences between genders, BMI groups, time spent sitting, and time spent exercising.

**Conclusion:**

The Italian version of SF-12v2 is a valid and reliable instrument to assess health-related quality of life among medical and health professionals' students.

## 1. Introduction

Quality of life (QOL) is a broad multidimensional concept that usually includes subjective evaluations of both positive and negative aspects of life. Health-related quality of life (HRQOL) includes those aspects of quality of life that can be shown to reflect either physical or mental health; it concerns physical, psychological, and social domains of health, and it is broadly accepted as an important outcome measure of health care [1].

The novel coronavirus SARS-CoV-2, responsible for the COVID-19 pandemic, led the various governments to implement strict domestic quarantine and social isolation policies that led people to significant psycho-emotional changes [2]. Regarding Italy, the Civil Protection Department adopted control measures such as information campaigns, closure of schools and universities, workplaces, and subsequently lockdown [3]. In addition to the morbidity and mortality due to the coronavirus-19 disease, the adopted response measures also had important physical and psychological consequences for individuals [4]. In fact, an outbreak is associated with considerable psychological disorders, such as depression, anxiety, and fear in specific communities and medical students [5, 6]. Alternatively, an increase in financial and family stress could be associated with some avoidance behaviors, which would have worsened their mental health and lead to a more passive lifestyle [7]. Sleep problems are also important public health issues when epidemics and disasters occur. This represents a common manifestation of anxiety and depression [8].

It is known that the presence of epidemics accentuates or creates new stressors, including fear and worry for oneself or loved ones, constraints on physical movement and social activities due to quarantine, and sudden and radical lifestyle changes. The unprecedented individual and societal fear and anxiety cause significant stress affecting HRQOL. Additionally, researchers have demonstrated a decrease in the level of outdoor physical activity and an increase in sedentary time during the COVID-19 pandemic as a result of home confinement. There is evidence of the current pandemic's psychological and mental health effects on students, who are known to be a vulnerable population [9]. A recent study indicated an increased percentage of Italian people with high and very high levels of distress compared to the European epidemiological statistics: 53.8% of respondents rated the psychological impact of the outbreak as moderate or severe, 16.5% reported moderate to severe depressive symptoms, 28.8% reported moderate to severe anxiety symptoms, and 8.1% reported moderate to severe stress levels [5]. It is known that the mental health of college students is significantly affected when faced with public health emergencies, and they require attention, help, and support of the society, families, and colleges [10, 11]. A study was conducted on Italian undergraduate students which showed 48.6% of the participants from the same student population decreased their physical activity; this result is in line with other researches performed with college students and adults from the United States and France [4].

Another study reported a decrease of physical activity in almost half of participants, where the majority of the students did not modify other habits, for example, diet and smoking habits [3]. A reduction in physical activity is known to contribute to stress levels, which is strongly associated with HRQOL [12].

In many studies, online questionnaires were used for the assessment of HRQOL and stress levels in students and general populations during the lockdown, and also, other surveys such as the World Health Organization Quality of Life- (WHOQOL-) BREF questionnaire [13] and the Pittsburgh Sleep Quality Index (PSQI) [14], many studies used the Perceived Stress Scale (PSS) [15].

The SF-12 Health Survey version 2 (SF-12v2) is a generic short-form health survey developed in the USA from the original SF-36. It produces two summary measures evaluating physical and mental self-perceived health; for this reason, it could be a suitable and complete tool to assess the self-perceived quality of life of university students because it allows investigating both the aspects. SF-12v2 has been successfully tested in several Western European countries on large samples of the general population, proving its brevity, comprehensiveness, reliability, validity, and cross-cultural applicability [16, 17]. Gandek et al. [18], in a cross-validation study, tested that the SF-12v2 suggested in the original United State study [19] for nine European countries (Denmark, France, Germany, Italy, the Netherlands, Norway, Spain, Sweden, and the United Kingdom). The SF-12v2, since then, has been extensively used in health status studies involving the general population [20–25] as well as in studies with disease groups. Until now, no study has investigated the physical and psychological effects on students during the outbreak due to COVID-19 using the SF-12v2.

The primary objective of this study is to evaluate the psychometric proprieties of the SF-12v2 in the assessment of self-perceived HRQOL of Italian students after the lifestyle changes due to the outbreak of COVID-19. The secondary objective is to detect different behaviors of the tool in different populations' groups in order to confirm the hypothesis that physical and mental health have been influenced by gender, university of origin (North, Central, and South Italy), body mass index, and number of hours spent sitting or exercising during the lockdown due to COVID-19 pandemic. Specifically, the authors hypothesized the following:

1. PSS would have had strong association with MCS12 and less strong with PCS12, as studied in previous studies [26, 27]
2. PCS12 and MCS12 scores would have had statistically different scores between people from northern/central/southern university, due to different rates of contagion and different restriction measures in these areas
3. PCS12 scores would have had highest for persons with normal BMI but lower for underweight and overweight as evaluated by Wee et al. [28]

## 2. Methods

A cross-sectional study was conducted by a research group of Sapienza University of Rome and Rehabilitation and Outcome Measures Assessment (ROMA) association to evaluate the psychometric properties of SF-12v2 health survey [29–39].

The institutional review board of Sapienza University of Rome approved the study and guaranteed ethical standards and procedures. The procedures followed the 1964 Helsinki declaration and its later amendments or comparable ethical standards. All individual participants included in the study were asked for informed consent. The datasets analyzed during the study are available from the corresponding author.

The study's inclusion criteria were to be university students attending the faculty of Medicine and Surgery or Health Professions in Italian Universities.

### 2.1. Outcome Measures

The SF-12v2 is a shortened version of SF-36v2; it is a self-reported outcome measuring HRQOL. It includes the same health domains as the SF-36 with fewer questions, making it a more practical instrument to evaluate physical and mental health. According to the original manual, it allows scoring of two health component measures: the Physical Component Summary (PCS12) and the Mental Component Summary (MCS12) [40]. The PCS12 and MCS12 are calculated by using US-derived item weights for response categories following recommendations from the authors of the instrument (which were done after having assessed the equivalence between country-specific and US weights in nine countries). Thus, each of the two health components is scored based on information from all 12 items, but different weights are applied for physical health and mental health, to achieve highest possible independence between the two health components.

The Perceived Stress Scale (PPS) is a 14-item scale used to measure perceived stress. In this study, the Italian version of PSS-10 was used; it is a shorter version of the original one and includes ten items (1, 2, 3, 6, 7, 8, 9, 10, 11, and 14), six negatively stated and four positively stated.

### 2.2. Procedures and Data Analysis

The research group recruited participants according to the inclusion criteria. A mailing list was used to recruit university students attending the faculty of Medicine and Surgery or Health Professions. All participants gave informed consent and then completed the SF-12v2 questionnaire.

### 2.3. Factor Structure

Previous studies [40] have found that 6 of the SF-12v2 items (1, 2, 3, 4, 5, 8) are most strongly associated with physical health, while 6 other items (6, 7, 9, 10, 11, 12) are most strongly associated with mental health. In order to confirm this result, we performed an exploratory factor analysis (EFA) with maximum likelihood factoring. Maximum likelihood and principal axis factoring are the generally recommended extraction methods [41]. Extracted factors were rotated by varimax rotation (orthogonal rotation method), and significant factor loading for EFA was set >4 [42]. The appropriateness of sampling was evaluated using the Keiser-Meyer-Olkin (KMO) and Bartlett's test of sphericity.

### 2.4. Reliability

The most appropriate measures of reliability of the PCS12 and MCS12 use reliability indices for weighted composite scores [43]. However, this approach is difficult, since it requires reliability estimates for each of the 8 subscales of the SF-12, some of which has only 1 item. Therefore, we estimated approximate reliability by using Cronbach's coefficient alpha for the 6 items most strongly associated with physical health and the 6 items most strongly associated with mental health. An alpha coefficient of 0.7 is usually seen as acceptable reliability.

Moreover, in conformity with previous studies on reliability of the tool, in order to evaluate Cronbach's alpha, we used the value of the response choice categories before weighting the indicator variables and computation of aggregate scores [23, 24, 44]. We reverse scored four items (1, 8, 9, and 10) so that a higher item value indicates better health for all Sf-12v2 items and summary scales.

### 2.5. Validity

The criterion validity was evaluated by comparing SF-12v2 values to PSS scores.

The two assessment tools were administered together, and the Pearson Correlation Coefficient (PCC) was calculated. PCC can be interpreted as follows: 0 indicates no linear relationship, +1/-1 indicates perfect positive/negative linear relationship, between 0 and ±0.3 weak relationship, between ±0.3 and ±0.7 moderate relationship, and between ±0.7 and ±1.0 strong relationship.

We also analyzed known group validity [45] through box plots and ANOVA analysis in order to investigate whether PCS12 or MCS12 differed as hypothesized between gender, BMI group, and number of hours spent sitting and exercising during the lockdown.

All statistical analyses were conducted using Statistical Package for Social Sciences (SPSS).

## 3. Results

The SF-12v2 questionnaire was administered together with the PSS to 583 medical and health professionals' students recruited by the Department of Human Neurosciences of Sapienza University of Rome in July 2020. The demographic characteristics of the sample are summarized in Table 1.

The results of the exploratory factor analyses are presented in Table 2. The Bartlett's test of sphericity has a *p* value < 0.001 and the KMO 0.863. This finding indicates that data were suitable for factor analysis. Factor analysis extracted 2 factors out of the 12 items that explained 54.82% of the variance. As shown in Table 2, factor 1 included 6 items (items 1, 2, 3, 4, 5, and 8), and factor 2 included 6 items (6, 7, 9, 10, 11, 12). These results confirm the allocation presented in the original manual [37].

Alpha coefficient was 0.69 as regards PCS12 of SF-12v2 and 0.84 as regards MCS12. From the “Cronbach's Alpha if Item Deleted” analyses, all items resulted to be necessary for the total subscale.

The correlation between the PSS and the MCS12 was -0.390 (*p* < 0.01), while the correlation with the PCS12 was 0.013.

The ANOVA analysis of known group validity is presented in Table 3 showing a statistically significant difference in MCS12 for gender, time spent sitting, and time spent exercising. A statistically significant difference was found also in PCS12 scores in people with different BMI.

Finally, Figures 1–10 show graphically through box plot differences in mean between PCS12 and MCS12 with time spent sitting and exercising, BMI, and Italian geographic area.

## 4. Discussion

The primary objective of this study was to evaluate the psychometric proprieties of the SF-12v2 in the assessment of self-perceived HRQOL of Italian students after the lifestyle changes due to the outbreak of COVID-19. The secondary objective was to detect different score levels of the tool in different populations' groups in order to confirm the hypothesis that physical and mental health are associated with gender, BMI, and number of hours spent sitting or exercising during the lockdown due to COVID-19 pandemic.

The results of this study showed that SF-12v2 is a reliable and valid tool to assess self-perceived HRQOL in Italian students in terms of internal consistency reliability and construct validity.

As hypothesized, we found a moderate linear correlation between the PSS scale and MCS12 but no correlation between PSS and PCS12 [26, 27].

We found statistically significant differences in scores of PCS12 in students with different BMIs showing that people with obesity had worse self-assessed physical health compared with those of people with normal weight [28].

As expected, scores of MCS-12 were lower for women than for men, but we did not find a similar difference for PCS12. This study shows statistically significant associations between MCS12 and time spent sitting and exercising during the lockdown due to COVID-19 pandemic. With regard to time spent sitting, lowest scores in the MCS12 were found for the group spending 12-18 hours sitting. Surprisingly, best MCS12 scores were seen in the group spending over 18 hours sitting. However, this may be a spurious result due to the small size of the group (*n* = 14). With regard to time spent exercising, highest MCS12 scores were seen in the groups spending 3 hours or more than 3 hours exercising. This means that students that did not practice physical activity during lockdown reported a lower level of mental health in SF-12v2. The mean value of the MCS12 (41.43) is about ten points below the norm score indicated for SF-12v2 [46]. This result is consistent with the current literature. In fact, other studies conducted on students found that while physical health was above the United States (US) general population norm (50 points), mental health scores of SF-12v2 were lower [47]. It has been demonstrated that increasing physical activity and promoting adequate sleep duration are key health promotion strategies for college students [48]. Regarding mental health, the literature shows increased symptoms of depression, anxiety, and stress related to COVID-19 due to psychosocial stressors such as life disruption, fear of illness, or fear of negative economic effects [49].

As regards the university location, we did not find statistically significant differences in HRQOL scores. North Italy was afflicted with COVID-19 first and has been in a stressful situation for the longest. However, this area had a better situation as regards hospitals and sanitary operation than other areas. Anyway, coronavirus spread rapidly also in the other areas [49].

## 5. Conclusion

This study showed that the Italian version of SF-12v2 is a valid and reliable instrument to assess HRQOL for medical and health professionals' students.

## Figures and Tables

**Figure 1:**
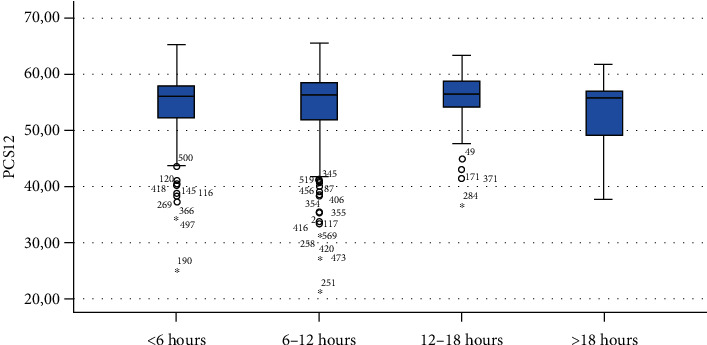
Correlation between Physical Component Summary (PCS12) of SF-12 and time spent sitting during the lockdown due to COVID-19 pandemic.

**Figure 2:**
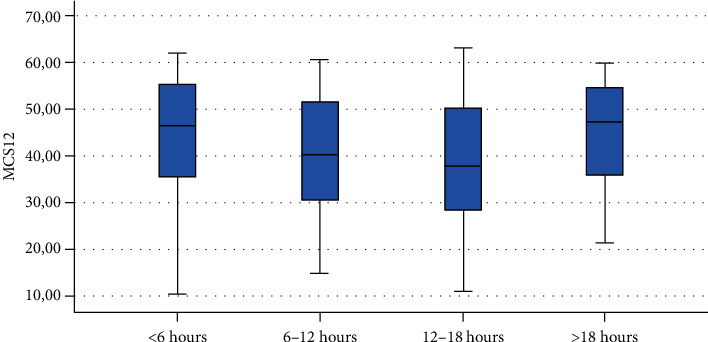
Correlation between Mental Component Summary (MCS12) of SF-12 and time spent sitting during the lockdown due to COVID-19 pandemic.

**Figure 3:**
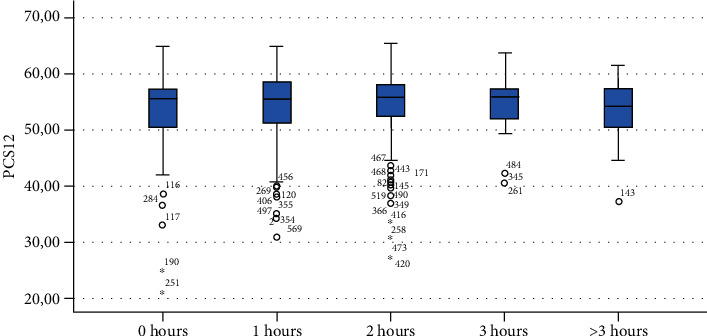
Correlation between Physical Component Summary (PCS12) of SF-12 and time spent exercising during the lockdown due to COVID-19 pandemic.

**Figure 4:**
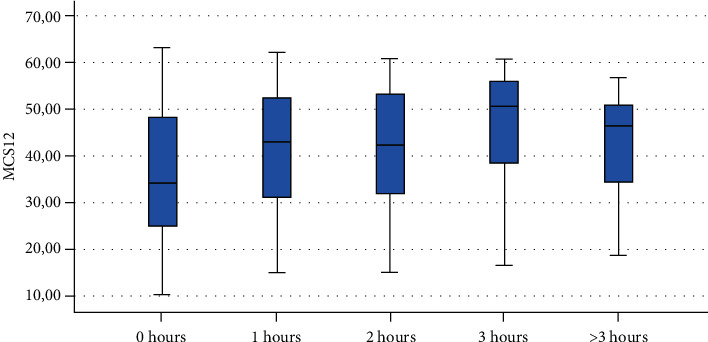
Correlation between Mental Component Summary (MCS12) of SF-12 and time spent exercising during the lockdown due to COVID-19 pandemic.

**Figure 5:**
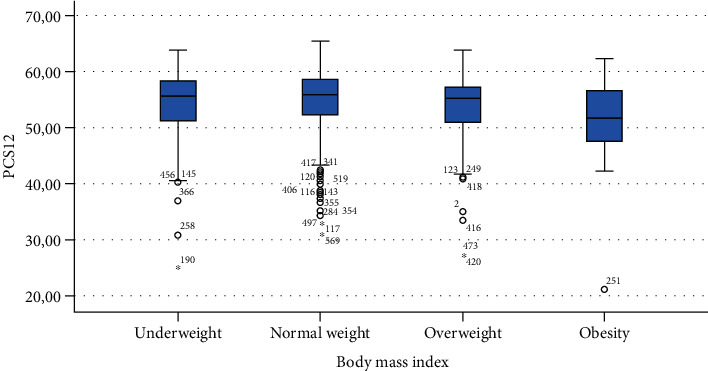
Correlation between Physical Component Summary (PCS12) of SF-12 and body mass index.

**Figure 6:**
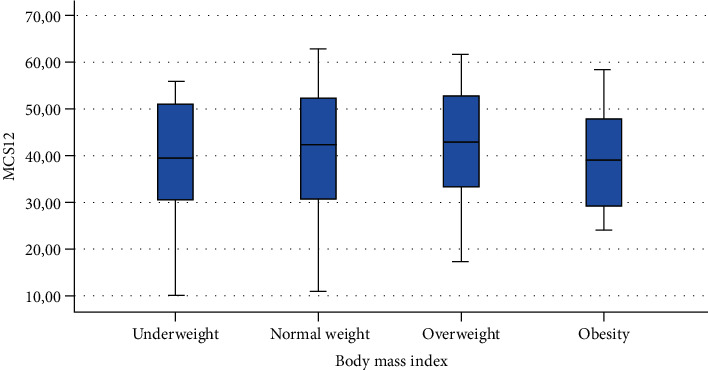
Correlation between Mental Component Summary (MCS12) of SF-12 and body mass index.

**Figure 7:**
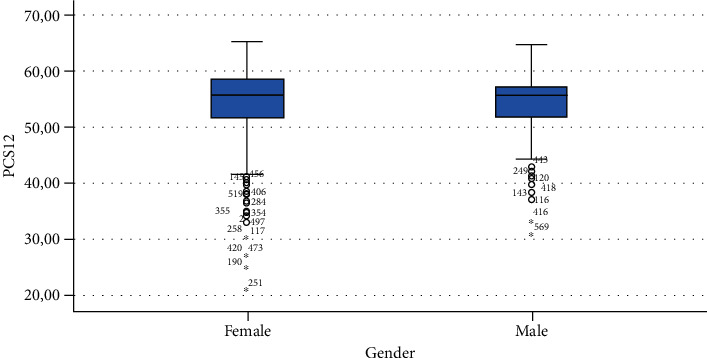
Correlation between Physical Component Summary (PCS12) of SF-12 and gender.

**Figure 8:**
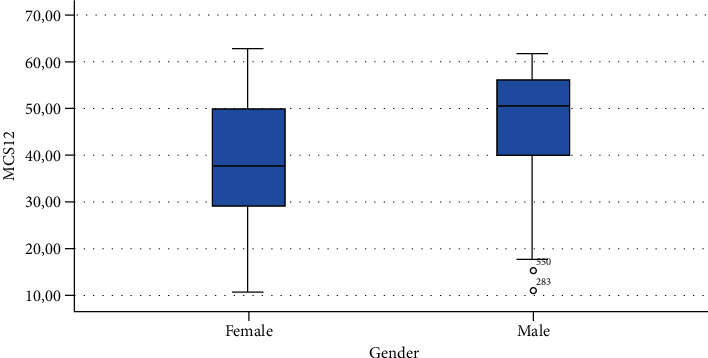
Correlation between Mental Component Summary (MCS12) of SF-12 and gender.

**Figure 9:**
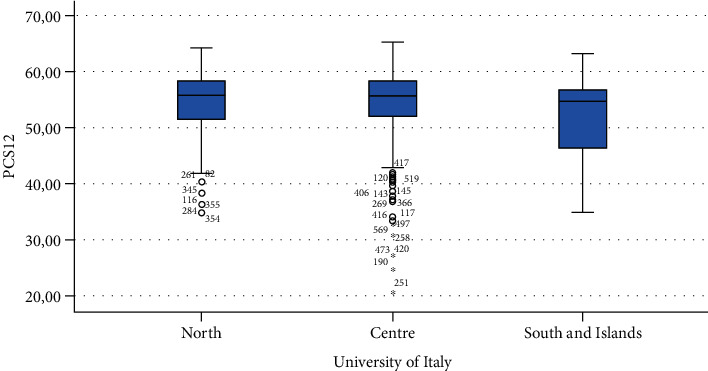
Correlation between Physical Component Summary (PCS12) of SF-12 and geographic area of Italy.

**Figure 10:**
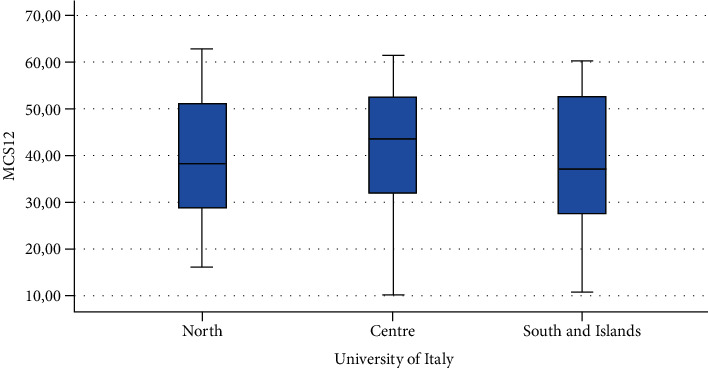
Correlation between Mental Component Summary (MCS12) of SF-12 and geographic area of Italy.

**Table 1:** Demographic characteristics of the sample.

	*N*° = 583
Age, mean ± SD	23.14 ± 5.17
Gender, *N*° (%)	
Female	404 (69.3)
Male	179 (30.7)
University, *N*° (%)	
North	134 (23)
Center	408 (70)
South and islands	41 (7)
Body mass index, *N*° (%)	
Underweight	47 (8.1)
Normal weight	422 (72.4)
Overweight	98 (16.8)
Obesity	16 (2.7)
Time spent sitting, *N*° (%)	
<6 hours	150 (25.7)
6-12 hours	383 (65.7)
12-18 hours	36 (6.2)
<18 hours	14 (2.4)
Time spent exercising, *N*° (%)	
0 hours	62 (10.6)
1 hours	290 (49.7)
2 hours	175 (30)
3 hours	34 (5.8)
>3 hours	22 (3.9)

**Table 2:** Exploratory factor analysis of SF-12v2 in Italian University students.

	MCS-12	PCS-12
Item 1	0.337	0.386
Item 2	0.055	0.705
Item 3	0.025	0.679
Item 4	0.220	0.687
Item 5	0.079	0.760
Item 6	0.724	0.144
Item 7	0.785	0.073
Item 8	0.266	0.712
Item 9	0.788	0.083
Item 10	0.792	0.183
Item 11	0.820	0.121
Item 12	0.695	0.222

**Table 3:** ANOVA analysis for SF-12v2 scores in Italian University students.

	*N*	PCS12 mean ± SD	*p*	MCS12 mean ± SD	*p*
Total	583	54.35 ± 6.61		41.43 ± 12.16	
Gender					
Female	404	54.36 ± 6.98	0.938	39.14 ± 11.82	0.001^∗^
Male	179	54.32 ± 5.71		46.62 ± 11.33	
University					
North	134	54.82 ± 5.99	0.740	39.81 ± 12.30	0.960
Center	408	54.41 ± 6.69		42.15 ± 11.91	
South and islands	41	52.16 ± 7.51		39.63 ± 13.72	
Body mass index					
Underweight	47	52.98 ± 8.45	0.050^∗^	39.99 ± 12.39	0.607
Normal weight	422	54.89 ± 6.04		41.40 ± 12.26	
Overweight	98	53.31 ± 7.12		42.56 ± 11.80	
Obesity	16	50.44 ± 9.37		39.67 ± 11.38	
Time spent sitting					
<6 hours	150	54.70 ± 6.18	0.727	43.88 ± 12.16	0.021^∗^
6-12 hours	383	54.20 ± 6.84		40.58 ± 11.93	
12-18 hours	36	54.90 ± 6.12		39.28 ± 13.17	
>18 hours	14	53.13 ± 6.4		44.08 ± 12.87	
Time spent exercising					
0 hours	62	52.86 ± 8.75	0.434	36.46 ± 13.61	0.007^∗^
1 hours	290	54.48 ± 6.18		41.75 ± 11.90	
2 hours	175	54.66 ± 6.79		41.90 ± 11.73	
3 hours	34	54.61 ± 5.1		45.09 ± 12.72	
>3 hours	22	53.89 ± 5.75		42.01 ± 10.89	

## Data Availability

The data used to support the findings of this study are available from the corresponding author upon request.
